# Implementing Green Supply Chain Management for Online Pharmacies through a VADD Inventory Model

**DOI:** 10.3390/ijerph16224454

**Published:** 2019-11-13

**Authors:** Yan-Kwang Chen, Fei-Rung Chiu, Yu-Cheng Chang

**Affiliations:** 1 Department of Distribution Management, National Taichung University of Science and Technology, Taichung 40401, Taiwan; 2 Department of Hotel and M.I.C.E Management, Overseas Chinese University, Taichung 40721, Taiwan; frchiu@ocu.edu.tw; 3 Department of Leisure and Recreation Management, Asia University, Taichung 41354, Taiwan; yuchen@asia.edu.tw

**Keywords:** online pharmacy, green supply chain management, visual-attention-dependent demand, sustainable inventory model, genetic algorithms

## Abstract

Online pharmacies are an important part of the modern healthcare system. They interact with customers through well-designed web interfaces to deliver the healthcare customers need. In addition to well-designed web interfaces, online pharmacies rely on an effective supply chain system to provide medical supplies and services, and especially effective inventory management for supply systems. As green supply chain management (GSCM) becomes increasingly considered by countries, how to develop a sustainable inventory model that takes into account the revenue growth of an online pharmacy while preventing waste and reducing energy costs has become very important. In line with this trend, the study develops a sustainable inventory model that focuses on both economic aspect (profit) and environmental aspect (losses from excessive inventory) within a framework of a single period multi-product inventory model. Specifically, the sustainable inventory model applies the visual-attention-dependent demand (VADD) rate to characterize customer demand in an online trading environment, thereby seeking a profitable marketing strategy and reducing losses due to excessive inventory. Since the complexity of model optimization will drastically increase due to the inclusion of many products in the problem, a Genetic Algorithm (GA) based solution procedure is proposed to increase the feasibility of the proposed model in solving real problems. The sustainable inventory model and the solution procedure are illustrated, compared, and discussed with an online pharmacy example. Additionally, a sensitivity analysis is formulated to study the influence of model parameters on the model solution, the loss of unsold inventory that results in a waste of resources and energy, and the profit of online pharmacies.

## 1. Introduction

E-commerce has been booming and has made great progress in bringing economic benefits to the healthcare industry. In the past few years, e-commerce technology provided clinics, hospitals, and doctors’ offices with an opportunity for telemedicine, such as Apps like Practo and FirstOpinion, and websites like Doctor2U and YourDoctors.Online. Under this system, the doctor provides text suggestions or has live chats with the patient without the need for a patient to visit a specialist or leave home. While this does not eliminate the need for face-to-face inspections, telemedicine can often help patients determine if an examination is needed [1]. Additionally, e-commerce technology provides opportunities for clinics, hospitals and doctors’ offices, and even customers, to streamline the purchase of medical equipment and supplies. There are two main business models in this area: Business-to-Business (B2B) and Business-to-Customer (B2C). B2B refers to a case where a business (medical device manufacturer) conducts a commercial transaction with another business (healthcare provider). An example of such a company is MedicalExpo, which offers an online exhibition of medical products that connects healthcare providers directly to medical device manufacturers around the world, allowing direct ordering and product delivery. In the B2C model, all transactions through a commercial website are made directly between the business (medical device manufacturer/healthcare provider) and the customers (patients). The customers visit the product catalogue of the site and place an order to purchase. After the business receives the order, it will deliver the products to the customer. Some online pharmacies such as Walgreens and Mycare.De are successful B2C cases [2]. 

Online pharmacies, internet pharmacies, and mail-order pharmacies have made tremendous headway in recent years. According to GLOBE NEWSWIRE [3], the global online pharmacy market was approximately USD 42.32 billion in 2018 and is expected to generate around USD 107.53 billion by 2025, at a Compound Average Growth Rate (CAGR) of around 14.26% between 2019 and 2025. With a booming online pharmacy market, how to increase online sales is one of the most important concerns of online pharmacies. Previous online sales research has shown that website interface design is critical to sales revenue. Of particular interest is the design of the product catalogue on the website. At the same time, online pharmacies rely heavily on effective and sustainable supply chains to deliver products and services. In particular, managing inventory and material flows is critical to achieving an effective and sustainable supply chain [4].

A pharmacy computerized inventory program that can optimize commodity availability and reduce commodity shortages would help online pharmacies use resources more effectively, preventing waste and reducing energy costs [5]. As green supply chain management (GSCM) has become increasingly considered, it is important to develop a sustainable inventory model that considers revenue growth, prevention of waste, and a reduction in energy costs for online pharmacies. The study aims to develop an inventory model that focuses on both economy (profit) and environmental development (loss from unsold inventory) for the online pharmacy, who sells multiple products through an online catalogue. This model is developed within a framework of a single period multi-product inventory model that allows the customer demand affected by price, product image size, and product image location (i.e., accounting for owners and cross-space effects). By this model, the optimal product image location, product price, and procurement quantity that maximize the profit can be efficiently determined. Since this model incorporates online customers’ browsing behavior into the demand function, this model could provide a useful analytical tool for online pharmacies when implementing GSCM. As multi-product is taken into account, the model optimization process is a Nondeterministic Polynomial (NP) hard. That is, when the number of products becomes large, it will take a lot of time to solve the problem. Facing the NP hard issue, a Genetic Algorithm (GA) based solution procedure is proposed with the model to increase the feasibility of the proposed model in practical problem solving.

The rest of this paper is organized as follows: The relevant literature is briefly reviewed in Section 2. The sustainable inventory model is developed in Section 3. Following this, a solution procedure based on GA is developed to optimize the product image placement, product price, and product procurement quantity that could maximize the profit of an online pharmacy with the environmental consideration in Section 4. In Section 5, the model and the solution procedure are illustrated and compared through an example, and a sensitivity analysis is made to recognize the impact of model parameters on the solution. In Section 6, the advantages, limitations, and research results of the model are discussed. Finally, conclusions are made in Section 7.

## 2. Literature Review

As we know, websites are the main bridges between online pharmacies and customers in the communication of products and services. Therefore, the information about products and services on the web page usually affects the decisions of customers to purchase, which in turn affects the inventory management policy of online pharmacies. However, the information about products and services on the webpage is quite diverse and complex. To simplify the complexity of the problem, the customer purchase decision here is assumed only subject to the visual attention on the product when browsing the webpage, as well as the impact of the product price. Accordingly, there are mainly two research streams related to our work, i.e., visual-attention-dependent demand and state-dependent inventory models in retail.

### 2.1. Visual-Attention-Dependent Demand

Visual attention usually refers to the choice of processing and focusing visual stimuli [6,7]. It manifests itself in the visible movement of the eyes and head, ensuring that the “spotlight” of attention illuminates the area of interest in the space. The “spotlight” of visual attention follows the scanning path on the stimulus, during which the eye briefly moves from one position to another with a short pause [8,9].

In the retail market, the customer’s visual attention is a requisite for a purchase [10]. Retailers recognize the importance of ensuring that the customer pays attention to their products and attempt to find a way to decide where to display products according to the channel format (in-store or online) [11]. The customer’s visual attention is usually affected by the type of product presented. When the customer observes a certain type of product, they will pay more attention to and actively seek product information, and even make some purchases cautiously [12,13]. This visual attention is called voluntary attention. The distribution of voluntary attention usually depends on the customer’s understanding of the search target, and the search process is top-down. On the other hand, involuntary attention is often driven by the stimuli in the customer’s field of vision [14,15,16,17], such as product color, size, shape, spatial arrangement, etc., and the search process is bottom-up [18]. When the customer is not clear about the search target, the website can more easily influence the viewer’s visual attention deployment by changing the visual stimulus. For example, giving an animated image can more easily attract the customer’s attention, while a larger image can give the viewer a deeper impression [19,20]. The first page receives more attention than the following pages [21]. Additionally, customers tend to focus on the left or top of the page [22] and often move along the zigzag path from the upper left to the lower right [23,24]. Chen et al. [25] synthesized the characteristics of visual attention in the literature to develop an economic model that optimizes the product image placement. Nevertheless, they did not discuss decisions related to the supply system and inventory.

### 2.2. State-Dependent Inventory Model

From a green supply chain perspective, in order to reduce the waste of resources and energy costs, it is important to decide how much inventory to keep and how much to order from the supplier. The economic order quantity (EOQ) model and the newsvendor model are two decision models commonly used to analyze inventory. Since the original models were proposed, many related studies have adopted different assumptions to match the actual situation. Among them, the inventory model with a state-dependent demand rate has received a lot of attention. Here, the state-dependent demand rate means that the demand is affected by some factors, including the way the products are displayed and the price of the products (see Table 1). 

Many scholars engaged in marketing research have observed that the sales at the retail level tend to be proportional to the facings of products displayed. Accordingly, they included the observations in their studies. For example, in the context of brick-and-mortar stores, Corstjens and Doyle [26] developed a shelf-space allocation model in which the demand rate is a function of shelf-space assigned to each product. Hwang et al. [27] extended Corstjens and Doyle’s model to determine the optimal product quantity on the shelf, order quantity, and product location. Hariga et al. [28] considered the effect of shelf space and display area location decisions on demand. For online retailing, the color, shape, size, and spatial arrangement of different product images are believed to influence the customer’s attention, which, in turn, affects their purchasing decisions [25]. According to this, Chen et al. [29] considered the product stored in its own warehouse and proposed a visual-attention-dependent demand (VADD) inventory model, where the demand rate is a function of the display area and size assigned to the product image. Chen et al. [30] extended Chen et al.’s model [29] to a drop-shipping environment.

Some research focused their attention on inventory models that incorporate a demand function with pricing decisions. In terms of single period inventory management issues, Whitin [31] firstly studied the price-setting newsvendor model for a single product. Petruzzi and Dada [32] and Agrawal and Seshadri [33] developed price-setting newsvendor models for multiple products, respectively. More recently, Murray et al. [34] proposed the price-setting newsvendor problem with resource capacity constraints for multiple products. In terms of multi-period inventory management issues, Urban and Baker [35] generalized the EOQ inventory model by formulating the demand as a function of price, time, and inventory level. Datta and Paul [36] analyzed the order-up-to models with stock-dependent and price-sensitive demand rates. You and Hsieh [37] analyzed the EOQ models with stock-dependent and price-sensitive demand rate. Avinadav et al. [38] designed the inventory model for deteriorating products with price- and time-dependent demands. Zhang et al. [39] and Lu et al. [40] developed an inventory model that incorporates a dynamic pricing strategy to the EOQ model for a deteriorating product in stock-dependent demand.

The aim of this study is to establish a sustainable inventory model for online pharmacies that allows customer demand to be influenced by customer’s visual attention and price. Optimization of this model will assist online pharmacies in determining the placement of items in the online catalogue, the price of the item, and the quantity of each item replenished. This research topic is similar to Chen et al. [29], but in Chen et al. [29], it is assumed that the products sold by the online retailer are non-deteriorating items and developed the model based on the multi-product EOQ framework. Considering that cosmeceuticals have a validity period, this sustainable inventory model is developed based on the single-period multi-product newsboy framework. In addition, this study considers the pricing of products, but Chen et al. [29] assumed that the price is known and fixed. At last, the sustainable inventory model focuses on both economic and environmental aspects. It differs from the existing literature that focuses only on the economic direction. 

## 3. The Sustainable Inventory Model

### 3.1. Problem Description

**Figure 1:**
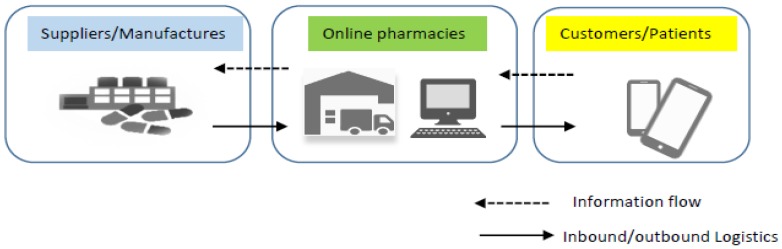
Pharmaceutical supply chain architecture.

**Figure 2:**
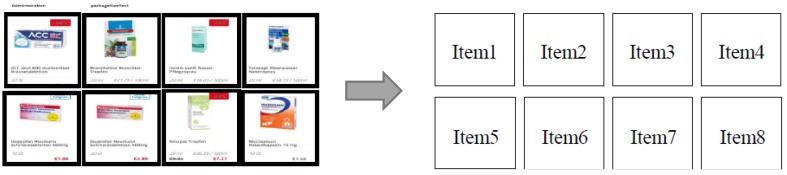
Configuration of product images on an online pharmacy catalogue.

Consider an online pharmacy procures products from a number of upstream suppliers and sells products to downstream customers through online catalogues (see Figure 1). The products displayed in an online catalogue are categorized by product category and displayed across multiple pages in a list or in an array (see Figure 2). For the convenience of description, it is assumed that there are N items to display on the product listing page of a certain product. The first page has S *×* T positions for product images while the following pages have V × W positions totally. Additionally, N = S *×* T *+* V × W. The product information contains the product images, names, and prices. Sometimes, in order to attract the attention of customers, the product image size on the first page is designed to be larger than that on other pages. The customer demand can be affected by price, product image size, and product image location (i.e., accounting for owners and cross-space effects). To present demand dynamics and practical reality in the context of retailer decision-making, the demand of each product is assumed stochastic at any time. The online pharmacy hopes to determine the price of the product and the configuration of the product image to create the highest revenue. At the same time, with the image configuration and price of each product, to determine the procurement quantity such that the loss of unsold products at the replenishment time could be reduced. The procurement decisions are made in the presence of upper and lower bounds on the procurement quantity of products. To simplify the complexity of the problem, this study assumed that the expiration dates of all products exceed the replenishment time, and the remaining products at the replenishment time will be bought back by the supplier at a price lower than the cost or sold at a discount. This residual value is called salvage value. The above “simplification” ensures that the products ordered during the replenishment cycle are not discarded due to expiration, so the order quantity for each replenishment can be fully supplied to the customers. Conversely, the products ordered during the cycle may be discarded due to expiration, so that only part of the order quantity can be supplied to the customers.

According to the above description, this study developed a sustainable inventory model to investigate the following research questions: First, taking the economic and environmental considerations into account, how should the product image on the online catalogue be placed? How to price the product Furthermore, how many pieces should be procured for each replenishment?

Second, comparing the above results and the two commonly used methods from the average price of the home products, the average procurement quantity per replenishment, the loss caused by the unsold inventory, and the total profit, which is better? Third, which model parameters can significantly affect the above results?

### 3.2. Nomenclature

Before the mode is derived, this section describes the symbols that the mode will use:

$\alpha_{i}$: the expected basic demand of product i.
$\beta_{ist}$: the space elasticity of product i when placed at the position (s, t) of the first page, ${0\;<\;\beta }_{ist}<\;1$.
$\beta_{\mathrm{ivw}}^{\text{’}}$: the space elasticity of product i when placed at the position (v, w) of the following pages, ${0\;<\;\beta }_{\mathrm{ivw}}^{\text{’}}<\;1$.
$\gamma_{\mathrm{ij}}$: the price cross-elasticity between products i and j. If i = j, then $\gamma_{\mathrm{ij}}<0$; otherwise, $\gamma_{\mathrm{ij}}\geq 0$.
A: the area of product image on the first page. $A\;\geq {\;A}^{\text{’}}>\;0$.
$A^{\text{’}}$: the area of product image on the following pages. $A^{\text{’}}$> 0.
$p_{i}$: the unit selling price of product i to be decided.
$x_{\mathrm{ist}}$: binary decision variable: $x_{\mathrm{ist}}$ = 1 if product i is placed at position (s, t) on the first page, otherwise $x_{\mathrm{ist}}$ = 0.
$y_{\mathrm{ivw}}$: binary decision variable: $y_{\mathrm{ivw}}$ = 1 if product i is placed at position (v, w) on the following pages, otherwise $y_{\mathrm{ivw}}$ = 0.
$x_{i}$: $x_{i}={(x}_{i11}{,\;\ldots ,\;x}_{i1T}{,\;x}_{i21}{,\;\ldots ,\;x}_{i2T}{,\;\ldots ,\;x}_{\mathrm{iS}1}{,\;\ldots ,\;x}_{\mathrm{iST}})$
$y_{i}$: $y_{i}={(y}_{i11}{,\;\ldots ,\;y}_{i1W}{,\;y}_{i21}{,\;\ldots ,\;y}_{i2W}{,\;\ldots ,\;y}_{\mathrm{iV}1}{,\;\ldots ,\;y}_{\mathrm{iVW}})$
p: $p={(p}_{1}{,\;p}_{2}{,\;\ldots ,\;p}_{N})$

### 3.3. The Demand Function

In order to present the dynamic nature of the demand, this study assumes that the demand of product i (i = 1, 2, ⋯, N) during each order cycle is stochastic and described by the following function:

(1) $$D_{i}\left(x_{i}{,y}_{i},p\right){\;=\;d}_{i}\left(x_{i}{,y}_{i},p\right){\;+\;\varepsilon }_{i}$$

where $d_{i}\left(x_{i}{,y}_{i}{,p}_{i}\right)$ represents the deterministic component of demand function. As described by Chen et al. (2016), the demand of online shoppers may be affected by product price, product image size, image location of the product itself, and the image locations of all other products. Therefore, the visual-attention-dependent demand is applied in the deterministic component of the demand function, as shown below:

(2) $$d_{i}\left(x_{i}{,y}_{i},p\right){\;=\;\alpha }_{i}\left\{\sum_{s=1}^{S}\sum_{t=1}^{T}{{(x}_{\mathrm{ist}}A)}^{\beta_{\mathrm{ist}}}+\sum_{v=1}^{V}\sum_{w=1}^{W}{{(y}_{\mathrm{ivw}}A^{\text{’}})}^{\beta_{\mathrm{ivw}}^{\text{’}}}\right\}\prod_{j=1}^{N}p_{j}^{\gamma_{\mathrm{ij}}}$$

This deterministic component contains three parts: the first part $\alpha_{i}$ denotes the basic demand of product *i.* The second part is composed of formulae $\sum_{s=1}^{S}\sum_{t=1}^{T}{{(x}_{\mathrm{ist}}A)}^{\beta_{\mathrm{ist}}}$ and $\sum_{v=1}^{V}\sum_{w=1}^{W}{{(y}_{\mathrm{ivw}}A^{\text{’}})}^{\beta_{\mathrm{ivw}}^{\text{’}}}$. The first formula denotes the effect of visual stimulation on the basic demand when product i is shown on the first page, while the second one denotes the effect of visual stimulation when the product is shown on the following pages. The third part $\prod_{j=1}^{N}p_{j}^{\gamma_{\mathrm{ij}}}$ in Equation (2) symbolizes the substitution effect between competitive products i and j on the basic demand. Here, $\gamma_{\mathrm{ii}}$ denotes the own-price elasticity of product i and ranges from −1 to −1.5, whereas $\gamma_{\mathrm{ij}}$ denotes the price cross-elasticity between the products i and j, and ranges from 0 to ${|\gamma }_{\mathrm{ii}}|\;/\;(N\;-\;1)$ [41].

The symbol $\varepsilon_{i}$ in Equation (1) represents the stochastic component of demand. As the expected demand $E(D_{i})$ is commonly defined to equal $d_{i}$ in the pricing literature, we chose $E(D_{i}{)\;=\;d}_{i}$ for its widespread use and that results in ${E(\varepsilon }_{i})\;=\;0$. Under the normally distributed uncertainty of demand, it can be assumed that: $\varepsilon_{i}\sim N(0,\;\sigma^{2}$).

### 3.4. The Objective Function

The objective of this model is to maximize the expected profit generated from the expected sale of products as well as the expected loss of unsold inventory. Thus, the objective function captures the economic and environmental considerations. 

First, let us look at the expected profit function of individual product i as follows.

(3) $$\sum_{d_{i}=0}^{q_{i}}\left[p_{i}d_{i}\;+{\;h}_{i}\left(q_{i}-d_{i}\right)-c_{i}q_{i}\right]\times \;\mathrm{Pr}\;\{D_{i}\left(x_{i}{,y}_{i},p\right){\;=\;d}_{i}\}+\sum_{d_{i}{=q}_{i}+1}^{\infty }\left(p_{i}-c_{i}\right)q_{i}\times \;\mathrm{Pr}\;\{D_{i}\left(x_{i}{,y}_{i},p\right){=\;d}_{i}\}$$

where $c_{i}$ represents the unit cost of product i; $d_{i}$ represents the demand of product i; $q_{i}$ represents the procurement quantity of product i; $h_{i}$ represents the salvage value for each unsold product i. The Formula (3) can be rewritten as:

(4) $$\left(p_{i}-c_{i}\right)q_{i}-\left(p_{i}-h_{i}\right)\sum_{d_{i}=0}^{q_{i}}\left(q_{i}-d_{i}\right)\times \;\mathrm{Pr}\;\{D_{i}\left(x_{i}{,y}_{i},p\right){=\;d}_{i}\}$$

If one denotes the cumulative distribution function (CDF) of demand for product i by $F_{i}\left(x_{i}{,y}_{i}{,p,d}_{i}\right)$, the Formula (4) can be simply expressed as:

(5) $$\left(p_{i}-c_{i}\right)q_{i}-\left(p_{i}-h_{i}\right)\sum_{d=0}^{q_{i}}F_{i}\left(x_{i}{,y}_{i}{,p,d}_{i}\right)$$

where $\left(p_{i}-c_{i}\right)q_{i}$ represents the revenue from product i, and $\left(p_{i}-h_{i}\right)\sum_{d=0}^{q_{i}}F_{i}\left(x_{i}{,y}_{i}{,p,d}_{i}\right)$ represents the loss of unsold product i.

Accordingly, the total profit of all products can be aggregated as:

(6) $$\prod \left(x,y,p,q\right)\;=\;\sum_{i=1}^{N}\left\{\left(p_{i}-c_{i}\right)q_{i}-\left(p_{i}-h_{i}\right)\sum_{d=0}^{q_{i}}F_{i}\left(x_{i}{,y}_{i}{,p,d}_{i}\right)\right\}$$

where x, y, and q are vectors of $x_{i}$, $y_{i}$, and $q_{i}$, respectively. 

In the current form of profit function, the decision variable $q_{i}$ appears in the range of summation and may cause challenges in the process of the model optimization. Facing this challenge, we modify the profit function with the following indicator variable $z_{i,d}$ [31]:

(7) $$z_{{i,d}_{i}}=\{\begin{array}{ll} 1 & {\mathrm{if}\;d}_{i}\;\leq {\;q}_{i}\\ 0 & {\mathrm{if}\;d}_{i}{\;>\;q}_{i} \end{array}$$

The modified profit function is expressed as:

(8) $$\begin{array}{c} \prod \left(x,y,p,q\right)=\sum_{i=1}^{N}\{\left(p_{i}-c_{i}\right)q_{i}-\left(p_{i}-h_{i}\right)\;\sum_{d_{i}=0}^{L_{i}}F_{i}\left(x_{i}{,y}_{i}{,p,d}_{i}\right)-(p_{i}\\ -h_{i})\sum_{d_{i}{=L}_{i}+1}^{U_{i}}F_{i}\left(x_{i}{,y}_{i}{,p,d}_{i}\right)z_{{i,d}_{i}}\} \end{array}$$

As we can see, the lower or upper bound in the range of summation is no longer a decision variable.

### 3.5. The Constraints

As the procurement decisions are made subject to the upper and lower bounds on the procurement quantity of products, as well as the match in product image to location, the constraints of the model are formulated as:

(9) $$L_{i}\;\leq {\;q}_{i\;}\leq {\;U}_{i}\;\forall \;i$$

(10) $$\sum_{s=1}^{S}\sum_{t=1}^{T}x_{\mathrm{ist}}+\sum_{v=1}^{V}\sum_{w=1}^{W}y_{\mathrm{ivw}}=\;1\;\;\;\;\;\;\;\;\;\forall \;i$$

(11) $$\sum_{i=1}^{N}x_{\mathrm{ist}}\;=\;1\;\;\forall \;s,\;t$$

(12) $$\sum_{i=1}^{N}y_{\mathrm{ivw}}\;=\;1\;\;\forall \;v,\;w$$

In Equation (9), $L_{i}$ represents the lower bound on $q_{i}$, $U_{i}$ represents the upper bound on $q_{i}$, and Equations (10)–(12) are the assignment constraints. Equation (10) indicates that each product image on the first page or the following pages is assigned to a single location. Equations (11) and (12) indicate that each location is only assigned to a single product image.

## 4. Model Optimization

As we can see from Equation (8), the modified profit function Π(x,y,p,q) is a function of demand parameter ($\alpha_{i}$,$\;\sigma_{i}$), cost parameters ($c_{i}$, $h_{i}$), space parameters ($A^{\text{’}}$, *A*, $\beta_{\mathrm{ist}}$, $\beta_{\mathrm{ivw}}^{\text{’}}$, $\gamma_{\mathrm{ij}}$), range parameters ($L_{i}$, $U_{i}$), and decision variables ($x_{\mathrm{ist}}$, $y_{\mathrm{ivw}}$, $p_{i}$, $q_{i}$). The optimization of the proposed model is to derive optimal image placement, price, and procurement quantity of the products that maximize the total profit, when given the values for basic demand, cost, space, and range parameters are known. Previous studies have pointed out that the product image configuration problem is the NP hard [26]. Since the decision variables of the above model include the product image configuration, the complexity of the model optimization is more complicated than the complexity of the product image configuration problem. In other words, it would be inefficient and time-consuming when solving the above model with traditional optimization methods for large scale problems.

Recently, several meta-heuristic algorithms such as Genetic Algorithms (GA) [42], Tabu search [43], Ant Colony Optimization (ACO) [44], Particle Swarm Optimization (PSO) [45], and Teaching-Learning-Based Optimization (TLBO) [46] have been proposed to find an approximate optimal solution to the NP hard problem. Since GA has been successfully applied to many combinatorial optimization problems, which are one type of NP hard problem [47,48], a GA-based solution procedure was developed to find the approximate optimal solution of the proposed model.

**Figure 3:**
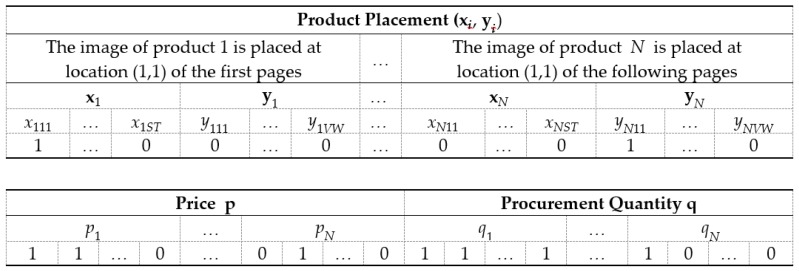
A chromosome for the decision variables pf proposed model.

GA is a global search and optimization technique motivated by the process of natural selection in a biological system [42,49]. When applying GA to model optimization, binary coding must be performed first for this problem. As shown in Figure 3, a potential solution (x, y, p, q) to the problem is expressed as a set of genes, which are linked together to form a chromosome.

The fitness value of the chromosome is evaluated by the modified profit function Π(x,y,p,q). If the chromosome fails to meet all model constraints, its fitness value will be set to zero. The algorithm for GA can be formulated as follows: first, GA randomly generates a set of chromosomes (i.e., population initialization), then this set of chromosomes will go through the selection, crossover, and mutation steps to find a chromosome with a superior fitness value that can propagate a new second-generation population to obtain the optimal solution. The termination condition is achieved when the number of generations is large enough or a satisfactory objective value is obtained. The pseudo code for the model optimization is shown in the Appendix A.

## 5. Application

In this section, the proposed model and solution process will be illustrated, compared, and discussed with the following example.

### 5.1. An Example

Consider a pharmacy that sells products through an online catalogue, which is divided into Beauty, Personal Care, Medicines and Treatments, Vitamins and Supplements, Sexual Wellness Supplements, and other categories. Each category is subdivided into several subcategories by the types of products. For the sake of illustration, the ‘Cold and Flu Medications’, which is a subcategory of ‘Cough, Cold and Flu’, is taken as an example to illustrate the application of the model and is discussed.

**Figure 4:**
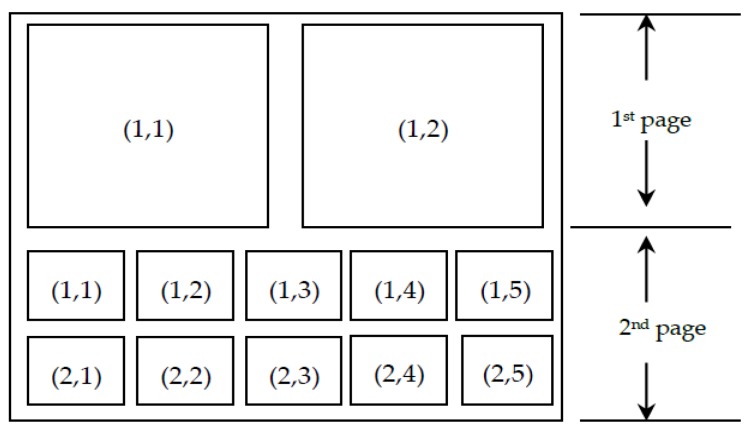
The configurable positions of the 12 product images.

This subcategory contains 12 kinds of cold and flu medicines, and these 12 product images are planning to be arranged in an array. The first page provides two locations with an area (A) of 12.96 (= 360 × 360 pixels/10,000) to place two product images, and the second page provides 10 locations with an area ($A^{\text{’}}$) of 1.44 (= 120 × 120 pixels/10,000) for the remaining product images. The configurable position of the product image is shown in Figure 4. The unit cost of these medicines ($c_{i}$) is between $2 and $4. The unit price ($p_{i}$) the pharmacy intends to set is ranged from 1.6 to 2.0 times of $c_{i}$. Through historical data, the manager observed the basic demand ($\alpha_{i}$) is between 55 and 75 products during each period, and the uncertainty of demand ($\varepsilon_{i}$) is normally distributed with a mean of $\mu_{i}\;=\;0$ and a standard deviation $\sigma_{i}$, which is about 10% of basic demand. Based on past experience, the salvage value ($h_{i}$) is approximately 25% of unit cost ($c_{i}$). Additionally, the space elasticity of the product shown on the first page ($\beta_{\mathrm{ist}}$) is between 0.35 and 0.40, while the space elasticity of the products shown on the following pages ($\beta_{\mathrm{ivw}}^{\text{’}}$) is between 0.20 and 0.32. Besides, all products are price sensitive. The price cross-elasticity of the product itself ($\gamma_{\mathrm{ii}}$) is about −1.375 and that between products ($\gamma_{\mathrm{ij}}$) is 0.125. The values of the aforementioned parameters are summarized in Table 2.

For each product, the amount of procurement is limited to the upper limit ($U_{i}$) of 100 supplies, while at least 50 of that limits the minimum required procurement quantity ($L_{i}$) according to contractual obligations to vendors.

In the above decision context, each product owns five allowable prices {1.6$c_{i},\;$1.7$c_{i}$, 1.8$c_{i}$, 1.9$c_{i}$, 2.0$c_{i}$} and 51 admissible procurement quantities of {50, 51, …, 100} to be chosen. Additionally, the combination of 12 configured product images equals 12! ($\approx 4{.79\;\times \;10}^{8}$). Therefore, there are about $3{.61\;\times \;10}^{37}$ candidate solutions in the solution space (as analyzed in Table 3). Due to the large size of this problem, the decisions on the price, product image configurations and procurement quantities are made through a GA based optimization procedure. As the quality of the solution generated by GA usually depends on the setting of their control parameters, it is necessary to set the control parameters of GA to obtain good results. The control parameters manipulated here are population size (PS) = 50, crossover probability (CP) = 0.10, mutation rate (MR) = 0.10, and number of generations (GN) = 1000. Table 4 shows the product image configuration, the prices, and the procurement quantities optimized by the proposed model. The result is compared to those of two methods commonly used in website design of most online pharmacies: ‘High-profit items first’ and ‘Best-seller items first’, which provide guidelines for product alignment. Given that, the price and procurement quantity of products for the two methods are determined to maximize the total profit.

From Table 4, the following findings can be obtained:

For the method ‘High-profit items first’, the first page is higher than the second one in terms of average prices.

For the method ‘Best-seller items first’, the products of the first page are higher than that of the second one in terms of procurement quantity.

In terms of the average price on the first page, the proposed model is between the methods of ‘High-profit items first’ and ‘Best-seller items first’.

The average procurement quantity obtained from the proposed model is greater than the average procurement quantity obtained from ‘High-profit items first’ or ‘Best-seller items first’.

In terms of the loss of unsold inventory, the proposed model is slightly higher than the method ‘High-profit items first’, but much lower than the method ‘Best-seller items first’.

The profit obtained from the proposed model is obviously better than those from the other two methods. The profit by the proposed model is 14.27% higher than that by ‘High-profit items first’ while 34.63% higher than that by ‘Best-seller items first’.

### 5.2. Sensitivity Analysis

In this section, we continue with the example in Section 5.1 to explore the effects of model parameters on the total profit, the loss of unsold inventory, and the model’s optimal solution by the experimental design and analysis of variance (ANOVA). In the experimental design, the factors were defined according to the model parameters, and each factor has two levels: low and high (see Table 5). Since each factor runs on two levels, this study examined the effects of these parameters on the total profit and the optimal solution obtained from the proposed model with a $2_{\mathrm{IV}}^{7-3}$ fractional factorial design that has resolution IV and 16 runs. The use of resolution IV can estimate the main effects unconfounded by two-factor interactions. At each run, the values of seven factors are taken as the $2^{7-3}$ fractional factorial design as shown in Table 6 and the total profit is maximized by the GA based solver. The average price of products on the first page, the average procurement quantity, the loss of unsold inventory, the total profit, and the products on the first page are also listed in Table 6 along with the corresponding total profit. Apparently, the products selected for placing on the first page are sensitive to the factors.

To further investigate the effects of factors on the average price of products on the first page, average procurement quantity, loss of unsold inventory, and total profit, an ANOVA was performed. The corresponding ANOVA results are presented in Table 7, Table 8, Table 9 and Table 10, respectively. Table 7 shows that the average price of the first page is significantly affected by the factor $\Delta \beta_{\mathrm{ivw}}^{’}/\beta_{\mathrm{ivw}}^{’}$. The factor $\Delta \beta_{\mathrm{ivw}}^{’}/\beta_{\mathrm{ivw}}^{’}$ has a positive effect, which implies changing space elasticity of the second webpage from low to high level increases the average price of the first page.

Table 8 shows the ANOVA result for the average procurement quantity. As we can see, the average procurement quantity is affected by factors $\sigma_{i}/\alpha_{i}$ and $\left(L_{i}{,\;U}_{i}\right)$. Both factors are observed to have negative effects, which implies changing the ratio of demand uncertainty to the average demand or changing the range of procurement quantity from low to high level decreases the average procurement quantity.

Table 9 presents the ANOVA on the loss of unsold inventory. The most significant factors are $\sigma_{i}/\alpha_{i}$ and $\Delta \beta_{\mathrm{ivw}}^{’}/\beta_{\mathrm{ivw}}^{’}$. A larger value of $\sigma_{i}/\alpha_{i}$ results in a larger loss of unsold inventory, whereas a large value of $\Delta \beta_{\mathrm{ivw}}^{’}/\beta_{\mathrm{ivw}}^{’}$ results in a smaller loss of unsold inventory.

Table 10 presents the ANOVA on the total profit. The most significant factors are $\sigma_{i}/\alpha_{i}$, $\Delta \beta_{\mathrm{ivw}}^{’}/\beta_{\mathrm{ivw}}^{’}$ and $\left(L_{i}{,\;U}_{i}\right)$. The larger value of $\sigma_{i}/\alpha_{i}$ and $\left(L_{i}{,\;U}_{i}\right)$ is a smaller value of total profit, whereas the large value of $\Delta \beta_{\mathrm{ivw}}^{’}/\beta_{\mathrm{ivw}}^{’}$ results in a larger total profit. 

## 6. Discussion

This study proposes a single period VADD inventory model for online pharmacies to cope with the sales growth of online marketing while reducing the cost of excessive inventory. The visual-attention-dependent demand function can be widely applied to various situations: First, the sizes of the product image of the first page and the following pages are allowed to be different. When this model is applied to the problem of the same product image size on each page, A =${\;A}^{’}$ can be made. Secondly, this model can be applied to highly or lowly involved items. As Flores et al. [9] said, customers often spend more visual attention on the product details for highly-involved items, and are less susceptible to the image size, image configuration, or price of the product. Conversely, customers pay less attention to the product details of lowly involved items, and are more affected by the image size, image placement, or the price of the product. When the displayed products are highly involved, the $\beta_{\mathrm{ist}}$ and $\beta_{\mathrm{ivw}}^{’}$values are close; on the contrary, when the products are of low involvement, the $\beta_{\mathrm{ist}}$ and $\beta_{\mathrm{ivw}}^{’}$ values are far apart. Thirdly, this model can be applied to problems with product images displayed across multiple pages in an array or in a list. When product images are displayed in a list, we can modify this model with T = 1 and W = 1. Finally, this model allows for price cross-elasticity between products. When the cross-elasticity between products does not exist, $\gamma_{\mathrm{ij}}$ = 0. This study does not address the estimation of price elasticity or spatial elasticity. For readers interested in how to estimate price elasticity or spatial elasticity, refer to Eisend [50].

The proposed model provides a link between the sales growth of online pharmacies and environmental sustainability. As compared with the methods of ‘High-profit items first’ and ‘Best-seller items first’, the model proposed can obtain more profits for online pharmacies. In the part of the loss caused by unsold inventory, the proposed model is much lower than ‘Best-seller items first’. This means that the proposed model can effectively reduce the loss of resources and energy costs and is conducive to environmental protection and sustainable development. Although the proposed model is slightly higher than ‘High-profit items first’ in terms of losses from unsold inventory, ‘High-profit items first’ emphasizes placing high-margin products on the first page. As Xu et al. [21] said, high profit also means higher price, so the price of the first page is usually higher than the price of other pages, which causes the viewers to be aware of the high price of the products sold in the pharmacy. In turn, this reduces the willingness to purchase and hurt the profits of online pharmacies.

From the sensitivity analysis results of the model parameters, the size of the demand variation will affect the procurement quantity of the products, the loss of unsold inventory, and the profit of online pharmacies. Since the profit function in the model only calculates the loss of unsold inventory and ignores the loss of inventory shortage, when the demand variability becomes larger, it will tend to reduce the procurement quantity of products to avoid the losses caused by excessive inventory. Even so, the large variation in demand means that the demand is difficult to estimate, and inevitably, the loss of unsold inventory will increase and damage the profits of online pharmacies. This result is in accordance with Chen et al. [30]. Next, the maximum and minimum quantity of products procured will also affect the quantity of products procured and the profit of online pharmacies. When the minimum procurement quantity is reduced, the optimal procurement amount will be reduced to avoid losses due to an excessive amount of inventory. When the range of products that can be procured is widened, the profit of online pharmacies naturally increases due to the large decision space of the procurement quantity. Lastly, the space elasticity of product images will affect the average price of the first page, the loss of unsold inventory, and the profit. When the space elasticity of all web pages is close or the same, it means that customers pay more attention to the product’s healthcare effect than price. In this case, it is not necessary to place the low-priced products on the first page to attract customers, so the price of the first page and the profits will increase. This result is again in accordance with Chen et al. [30]. This study developed a GA based solution procedure to solve the models. The GA based solution procedure is able to handle the model optimization. However, GA is generally characterized by the control parameters, so these parameters should be tuned for efficiency. An experimental design may be helpful in the setting of the control parameters to reduce the search time and improve solution quality, interested readers can refer to Grefenstette [51].

## 7. Conclusions

E-commerce technologies have provided modern pharmacies with opportunities to simplify sales. When pharmacies sell products through online product catalogues, the design of online catalogues and pricing often affect the revenue of product sales and are closely related to the supply and inventory of products. This study has developed a single period, multiproduct inventory model that considers the revenue of an online pharmacy and waste of resources caused by excessive inventory. Optimization of this model can provide online pharmacies with the best solution for online catalogue design, product pricing and procurement quantity. This model can help online pharmacies generate more profit than the most commonly used methods: ‘High-profit items first’ and ‘Best-seller items first’ in online pharmacies, while reducing the waste of resources and energy caused by excessive inventory. In the current society that increases emphasis on environmental resources and sustainable development, this model provides a means to achieve the goal of ‘green growth’. The sensitivity analysis has been made to determine the model parameters that could significantly affect the solution results. Analytical results show that ‘demand variation’, the ‘space elasticity’, and the ‘the maximum and minimum quantity of products procured’, could affect the model solution, the loss of unsold inventory, and the total profit. Therefore, it should be more cautious than other model parameters when using the values of these parameters.

Compared to traditional online stores, as the health care system pays more attention to meeting the needs of customers as much as possible, an interesting line of future research can try to incorporate the loss of stock-outs into the model, making the proposed model more applicable to the health care system. Another interesting line of research would be to focus on the estimation of price elasticity and space elasticity of various supplies to expand the applicability of the model.

## Figures and Tables

**Table 1:** Some inventory models with state-dependent demands.

Source	Inventory Model	Demand Rate Dependent on	Channel Format	Pricing?	Multiple Products?
Corstjens and Doyle [26]	EOQ	Stock	Off-line	No	No
Hwang et al. [27]	EOQ	Location and stock	Off-line	No	Yes
Hariga et al. [28]	EOQ	Stock	Off-line	No	Yes
Chen et al. [29]	EOQ	Visual attention	On-line	No	Yes
Chen et al. [30]	EOQ	Visual attention	On-line	No	Yes
Whitin [31]	Newsvendor	Price	Off-line	Yes	No
Petruzzi and Dada [32]	Newsvendor	Price	Off-line	Yes	No
Agrawal and Seshadri [33]	Newsvendor	Price	Off-line	Yes	No
Murray et al. [34]	Newsvendor	Price	Off-line	Yes	Yes
Urban and Baker [35]	EOQ	Stock, price, and time	Off-line	Yes	No
Datta and Paul [36]	Order-up-to	Stock and price	Off-line	Yes	No
You and Hsieh [37]	EOQ	Stock and price	Off-line	Yes	No
Avinadav et al. [38]	EOQ	Price and time	Off-line	Yes	No
Zhang et al. [39]	EOQ	Stock and price	Off-line	Yes	No
Lu et al. [40]	EOQ	Stock and price	Off-line	Yes	No

**Table 2:** Parameter values for the twelve merchandises.

Product (i)	$\alpha_{i}\;\text{\$}/\mathrm{Unit}$	$c_{i}\;\text{\$}/\mathrm{Unit}$	$h_{i}\;\text{\$}/\mathrm{Unit}\text{-}\mathrm{Year}$	$1\mathrm{st}\;\mathrm{Page}\;\beta_{\mathrm{ist}}$		$2\mathrm{nd}\;\mathrm{Page}\;{\beta \text{’}}_{\mathrm{inw}}$									
				(1,1)	(1,2)	(1,1)	(1,2)	(1,3)	(1,4)	(1,5)	(2,1)	(2,2)	(2,3)	(2,4)	(2,5)
1	74.00	2.1	0.53	0.367	0.352	0.304	0.301	0.286	0.272	0.272	0.252	0.216	0.204	0.203	0.202
2	70.00	2.4	0.60	0.367	0.352	0.304	0.301	0.286	0.272	0.272	0.252	0.216	0.204	0.203	0.202
3	59.00	3.8	0.95	0.367	0.352	0.304	0.301	0.286	0.272	0.272	0.252	0.216	0.204	0.203	0.202
4	62.00	3.3	0.83	0.367	0.352	0.304	0.301	0.286	0.272	0.272	0.252	0.216	0.204	0.203	0.202
5	70.00	2.4	0.60	0.367	0.352	0.304	0.301	0.286	0.272	0.272	0.252	0.216	0.204	0.203	0.202
6	74.00	2.1	0.53	0.367	0.352	0.304	0.301	0.286	0.272	0.272	0.252	0.216	0.204	0.203	0.202
7	75.00	2.1	0.51	0.367	0.352	0.304	0.301	0.286	0.272	0.272	0.252	0.216	0.204	0.203	0.202
8	64.00	3.1	0.78	0.367	0.352	0.304	0.301	0.286	0.272	0.272	0.252	0.216	0.204	0.203	0.202
9	69.00	2.5	0.63	0.367	0.352	0.304	0.301	0.286	0.272	0.272	0.252	0.216	0.204	0.203	0.202
10	65.00	2.9	0.73	0.367	0.352	0.304	0.301	0.286	0.272	0.272	0.252	0.216	0.204	0.203	0.202
11	58.00	3.9	0.98	0.367	0.352	0.304	0.301	0.286	0.272	0.272	0.252	0.216	0.204	0.203	0.202
12	63.00	3.2	0.80	0.367	0.352	0.304	0.301	0.286	0.272	0.272	0.252	0.216	0.204	0.203	0.202

**Table 3:** Analysis of candidate solutions to the 12-product problem.

Problem Size	Position Combinations	Price Combinations	Procurement Quantity Combinations	Total Combinations
N = 12, |p| = 5, |q| = 51	$\text{\textasciitilde{}}4{.79\;\times \;10}^{8}$	$\text{\textasciitilde{}}2{.44\;\times \;10}^{8}$	$\text{\textasciitilde{}}3{.09\;\times \;10}^{20}$	$\text{\textasciitilde{}}3{.61\;\times \;10}^{37}$

**Table 4:** Comparisons of results from the proposed approach and two commonly used methods.

Pages	Positions	Proposed Model			‘High-Profit Items First’			‘Best-Seller Items First’		
		Item (i)	$p_{i}$	$q_{i}$	Item (i)	$p_{i}$	$q_{i}$	Item (i)	$p_{i}$	$q_{i}$
1st page	(1,1)	12	6.31	99	11	6.70	67	7	4.11	98
	(1,2)	9	5.03	97	3	6.81	75	1	4.21	99
2nd page	(1,1)	4	6.58	50	4	6.26	50	3	6.81	51
	(1,2)	11	7.09	51	12	5.99	50	6	4.23	88
	(1,3)	3	7.19	51	8	5.59	54	5	4.83	62
	(1,4)	10	5.83	52	10	5.83	52	2	4.85	62
	(1,5)	1	4.21	94	9	4.53	75	9	5.03	61
	(2,1)	8	6.22	50	2	4.60	75	10	5.25	55
	(2,2)	7	4.11	93	5	4.34	53	8	6.22	51
	(2,3)	6	4.23	93	1	4.21	75	12	6.31	50
	(2,4)	2	4.12	70	6	4.23	75	4	5.27	62
	(2,5)	5	4.83	72	7	4.11	90	11	7.49	50
Average price (1st page)			5.67			6.76			4.16	
Average procurement quantity				73			66			66
Loss of unsold inventory (Its share of revenue)		$178.00 (8%)			$101.00 (5%)			$417.00 (21%)		
Total profit		$2062.96			$1805.33			$1532.34		

**Table 5:** Factors and Levels in the experimental design.

Factors	Parameters	Level	
		Low (−1)	High (+1)
A	$\sigma_{i}/\alpha_{i}$	0.1	0.3
B	$h_{i}/c_{i}$	0.1	0.25
C	$\Delta \beta_{\mathrm{ist}}/\beta_{\mathrm{ist}}$	1.0	1.5
D	$\Delta \beta_{\mathrm{ivw}}^{’}/\beta_{\mathrm{ivw}}^{’}$	1.0	1.5
E	$\Delta \gamma_{\mathrm{ij}}/\gamma_{\mathrm{ij}}$	0.8	1.0
F	$A/A^{’}$	1	9
G	$\left(L_{i}{,\;U}_{i}\right)$	(50,100)	(25, 125)

**Table 6:** Values for factors and responses of factional factorial design.

	Factors								Responses			
Runs	A	B	C	D	E	F	G	R1	R2	R3	R4	R5
1	−1	−1	−1	−1	−1	−1	−1	5.67	73	6%	2134.06	{12,9}
2	1	−1	−1	−1	1	−1	1	7.04	35	19%	888.65	{4,11}
3	−1	1	−1	−1	1	1	−1	5.67	73	8%	2062.96	{12,9}
4	1	1	−1	−1	−1	1	1	5.89	40	19%	967.74	{7,11}
5	−1	−1	1	−1	1	1	1	4.36	61	5%	1725.67	{7,9}
6	1	−1	1	−1	−1	1	−1	5.29	57	18%	1459.03	{10,9}
7	−1	1	1	−1	−1	−1	1	6.11	68	2%	2029.08	{3,9}
8	1	1	1	−1	1	−1	−1	5.29	58	21%	1443.65	{10,9}
9	−1	−1	−1	1	−1	1	1	6.30	67	2%	2010.54	{3,9}
10	1	−1	−1	1	1	1	−1	6.90	52	11%	1525.67	{12,11}
11	−1	1	−1	1	1	−1	1	6.30	68	3%	2048.44	{3,9}
12	1	1	−1	1	−1	−1	−1	7.72	51	6%	1625.38	{3,11}
13	−1	−1	1	1	1	−1	−1	7.09	71	3%	2275.35	{12,11}
14	1	−1	1	1	−1	−1	1	5.40	45	15%	1190.87	{4,1}
15	−1	1	1	1	−1	1	−1	7.09	73	2%	2383.58	{12,11}
16	1	1	1	1	1	1	1	6.21	46	20%	1219.91	{4,10}

A: $\sigma_{i}/\alpha_{i}$; B: $h_{i}/c_{i}$; C: $\Delta \beta_{\mathrm{ist}}/\beta_{\mathrm{ist}}$ D: $\Delta \beta_{\mathrm{ivw}}^{’}/\beta_{\mathrm{ivw}}^{’}$; E: $\Delta \gamma_{\mathrm{ij}}/\gamma_{\mathrm{ij}}$; F: $A/A^{’}$; G: $\left(L_{i}{,\;U}_{i}\right)$. R1: Average price of the first page; R2: Average procurement quantity; R3: Loss of unsold inventory (share of revenue); R4: Total profit; R5: Products displayed on the first page.

**Table 7:** The output of ANOVA for the average price of products on the 1st page.

Source	R-Square = 0.3239 Adjusted R-Square = 0.2756 F = 6.71 Pr > F = 0.0214 *					
	Stdized Effect	Sum of Squares	df	Mean Square	F Value	Pr > F
$\Delta \beta_{\mathrm{ivw}}^{’}/\beta_{\mathrm{ivw}}^{’}$	0.96	3.70	1	3.70	6.71	0.0214 *
residual error		7.71	14	0.55		
total		11.41	15			

* significance level 0.05

**Table 8:** The output of ANOVA for the average procurement quantity.

Source	R-Square = 0.9188 Adjusted R-Square = 0.9063 F = 73.54 Pr > F = 0.0001 *					
	Stdized Effect	Sum of Squares	df	Mean Square	F Value	Pr > F
$\sigma_{i}/\alpha_{i}$	−21.25	1806.25	1	1806.25	121.51	0.0001 **
$\left(L_{i}{,\;U}_{i}\right)$	−9.75	380.25	1	380.25	25.58	0.0002 **
residual error		193.25	13	14.87		
total		2379.75	15			

* significance level 0.05; ** significance level 0.01.

**Table 9:** The output of ANOVA for the loss of unsold inventory.

Source	R-Square = 0.8268 Adjusted R-Square = 0.8001 F = 31.02 Pr > F = 0.0001 **					
	Stdized Effect	Sum of Squares	df	Mean Square	F Value	Pr > F
$\sigma_{i}/\alpha_{i}$	0.12250	0.060025	1	0.060025	54.66	0.0001 **
$\Delta \beta_{\mathrm{ivw}}^{’}/\beta_{\mathrm{ivw}}^{’}$	−0.04500	0.008100	1	0.008100	7.38	0.0180 *
residual error		0.014275	13	0.001098		
total		0.082400	15			

* significance level 0.05; ** significance level 0.01.

**Table 10:** The output of ANOVA for the total profit.

Source	R-Square = 0.9655 Adjusted R-Square = 0.9569 F = 112.03 Pr > F = 0.0001 **					
	Stdized Effect	Sum of Squares	df	Mean Square	F Value	Pr > F
$\sigma_{i}/\alpha_{i}$	−793.6	2,519,188	1	2,519,188	266.83	0.0001 **
$\Delta \beta_{\mathrm{ivw}}^{’}/\beta_{\mathrm{ivw}}^{’}$	196.1	1,583,840	1	1,583,840	16.29	0.0020 **
$\left(L_{i}{,\;U}_{i}\right)$	−353.6	500,125	1	500,125	52.97	0.0001 **
residual error		113,295	12	9441		

** significance level 0.01.

## Appendix A

The pseudo code for the model optimization (modified from [30])

-------------------------------------------------------------------------------------------

Input:

- demand parameter $\alpha_{i}$,$\;\sigma_{i}$
- cost parameters $c_{i}$, $h_{i}$
- space parameters: $A^{’}$, *A*, $\beta_{\mathrm{ist}}$, $\beta_{\mathrm{inw}}^{’}$, $\gamma_{\mathrm{ij}}$
- min-max ranges placed on each of the decision variables: $L_{i}$, $U_{i}$

Output: solution S = ($x_{\mathrm{ist}}$, $y_{\mathrm{ivw}}$, $p_{i}$, $q_{i}$)

-------------------------------------------------------------------------------------------

Begin;

//Initialization

Generate PS chromosomes (feasible solutions) randomly;

Save them in the population Pop;

//Loop until the terminal condition

For k=1 to number of generation (GN) do

{

//Evaluate Pop

Calculate the fitness of chromosomes in population Pop;

Set fitness=Π(x, y, p, q) by Equation (8) if the chromosome meets all model constraints (9)–(12); Otherwise, set fitness = 0;

//Elitism based selection

Select (1 − CP) × PS members from current population Pop by

“Roulette Wheel” method and save to Pop1;

//Crossover

Select CP × PS members from population Pop;

Pair them up;

Generate offsprings by “two-point” crossover scheme;

Save to Pop1;

//Mutation

Select MR × PS members from population Pop1;

Invert a randomly selected bit in each;

Save to Pop1;

Pop = Pop1;

}

//Increment

k = k + 1;

Return the fittest member from Pop.
